# Culturally Safe Neonatal Care: Talking With Health Practitioners Identified as Champions by Indigenous Families

**DOI:** 10.1177/10497323231164550

**Published:** 2023-03-23

**Authors:** Anna Adcock, Fiona Cram, Liza Edmonds, Beverley Lawton

**Affiliations:** 1 Te Tātai Hauora o Hine National Centre for Women’s Health Research Aotearoa, 8491 Victoria University of Wellington, Wellington, New Zealand; 2 Katoa Ltd., Auckland City, Auckland, New Zealand; 3 Dunedin Hospital, Southern District Health Board, Dunedin, New Zealand; 4 Kōhatu Centre for Hauora Māori, Division of Health Sciences, 93760 University of Otago, Dunedin, New Zealand

**Keywords:** New Zealand, Māori/Indigenous, neonatal intensive care, tertiary care, preterm/premature birth, cultural safety, champions, qualitative research, Kaupapa Māori research, perinatal care

## Abstract

The burden of health inequities borne by Indigenous peoples can be overwhelming, especially when mothers and newborns’ lives are at stake and health services seem slow to invest in responsiveness. In Aotearoa (New Zealand), urgent action is required to eliminate persistent systemic inequities for Māori (Indigenous) whānau (family collectives that extend beyond the household). This Kaupapa Māori (by Māori, for Māori) qualitative study aimed to explore the views of health practitioners identified as champions by whānau of preterm Māori infants. Ten health practitioners were interviewed and asked about their involvement with the whānau, their role in explanations and communication, and their thoughts on whānau coping. Interview data were analysed using interpretative phenomenological analysis. Three superordinate themes were identified: working together in partnership, a problem shared is a problem halved, and sacred space. Collaboration between health practitioners and with whānau was important to the champions and central to their goal of enabling whānau autonomy. This was built on a foundation of connectivity, relationships, and a full appreciation that childbirth is a sacred time that is potentially disrupted when an infant is born prematurely. The values- and relationship-based practices of these champions protected and uplifted whānau. They showed that health practitioners have important roles in both the elimination of inequities and the sustaining of Māori self-determination. This championship is an exemplar of what culturally safe care looks like in day-to-day practice with Māori and is a standard that other health practitioners should be held to.

## Introduction

In Aotearoa (New Zealand), as in other high-resource settler-colonial nation-states, Māori (Indigenous) women and infants are at increased risk of adverse perinatal outcomes (Edmonds et al., 2021; Hickey et al., 2021; PMMRC, 2021; Smylie et al., 2010). These include higher rates of preterm birth (<37 weeks gestation) and its associated long-term sequelae such as disability and cerebral palsy, higher rates of mortality (death) for infants and mothers, and higher rates of missing data and likely missing care (Edmonds et al., 2021). Although maternity and infant care in Aotearoa are funded, these inequities persist and are linked to structural determinants of health, such as poverty and racism (PMMRC, 2022). Both structural determinants and Māori health inequities are indicators of the failure of the Crown (and successive governments) to fulfil their obligations set out in Te Tiriti o Waitangi (Te Tiriti) (an 1840 treaty that paved the way for colonial settlement) – including the guarantee of Māori tino-rangatiratanga (autonomy, self-determination, sovereignty) (Waitangi Tribunal, 2019).

An abundance of research evidences the structural, institutional, and interpersonal racism that Māori face in the health system, and the negative impacts of this on Māori health and wellbeing (Cormack et al., 2018; Graham & Masters-Awatere, 2020; Palmer et al., 2019; Thayer et al., 2019). When experiencing an adverse perinatal event, such as preterm birth, Māori whānau (family collectives that extend beyond the household) have reported that maternal and neonatal hospital environments, for example, neonatal intensive care units (NICUs), are not always culturally safe, responsive, supportive of diversity, or accommodating of Māori cultural protocols (Adcock et al., 2021, 2022; Māori and Indigenous Analysis Ltd. & Rautaki Ltd, 2010; Stevenson, Cram, et al., 2020; Thompson, 2009). Adding further stress, over one-third of women/infants will have to be transferred away from their home for access to a NICU or other appropriate care (PMMRC, 2021). This is isolating and disruptive for whānau (Stevenson, Cram, et al., 2020).

Current models of perinatal care that perpetuate inequities for Māori require racism to be challenged and eliminated (Dawson et al., 2022; Edmonds et al., 2021). As well as imploring the government and health sector to address structural and institutional racism, the Perinatal and Maternal Mortality Review Committee (PMMRC) (2022) has recently called for regulatory bodies to mandate cultural safety training for the maternity and neonatal workforce as part of this important anti-racist agenda. Cultural safety requires care that recognises and respects, rather than ignores, difference (Ramsden, 2015). It puts the obligation on health practitioners to do the relational work and examine power dynamics. They must recognise and address biases/prejudices – championing critical consciousness through accountability and reflexivity (Curtis et al., 2019).

Perinatal care that champions cultural safety as a fundamental requirement has the potential to improve perinatal outcomes (Kildea et al., 2021; Marriott et al., 2019; Tipene-Leach & Abel, 2019; Wodtke et al., 2022). For example, an Indigenous model of Birthing on Country in Australia emphasises the importance of relationships, with culturally safe care an integral part of best practice medical care (Kildea et al., 2021, 2019). Pregnant women who received this model of care were less likely to have a preterm birth (Kildea et al., 2021, 2019). While there have been no such Indigenous maternity models to reduce preterm birth in Aotearoa, mainstream holistic models Family Centred Care (FCC) and Family Integrated Care (FiCare) have been utilised in NICUs to involve and empower parents/guardians in infant cares (O'Brien et al., 2018; Serlachius et al., 2018), and have been shown to improve infant outcomes and parental wellbeing and satisfaction (Ding et al., 2019; O'Brien et al., 2018). These models are based on non-Indigenous ideas of family (McCalman et al., 2017), so a question remains about their appropriateness for Māori whānau. It may be that the delivery of these models has been or can be tailored by culturally safe health practitioners who champion whānau-centred care.

Stevenson and colleagues (2020) have created a framework called Te Hā o Whānau as a call for Māori whānau voices to lead culturally safe and Te Tiriti compliant maternal–infant health care. Health practitioners have an important role in this – working in partnership with whānau to support their autonomy/self-determination (Stevenson, Filoche, et al., 2020). Culturally safe care, involving meaningful communication and shared decision-making between whānau and health practitioners, is key to facilitating positive NICU experiences (Adcock et al., 2021; Māori and Indigenous Analysis Ltd. & Rautaki Ltd, 2010; Stevenson, Cram, et al., 2020; Thompson, 2009). Recognising and involving whānau, as collectives that go beyond nuclear notions of family, is important for holistic care (Masters-Awatere et al., 2019; Wilson et al., 2021), and may go some way to mitigating some of the stresses associated with preterm birth (Adcock et al., 2021).

Champions of culturally safe neonatal care, both Māori and non-Māori, are vital to affecting the changes called for by the PMMRC (2022) and others (Dawson et al., 2022; Edmonds et al., 2021). They have been identified as playing an important role in the training and delivery of culturally safe care (Doran et al., 2019; Petric et al., 2022). However, literature exploring the values and practices of champions of culturally safe care is limited. One qualitative study in Canada reported the views of health practitioners identified as champions of cultural safety by Aboriginal Patient Navigators (Foster, 2017). The findings suggest that developing good relationships – showing respect, being non-judgemental, and providing client-centred care – is crucial for cultural safety. They also found significant organisational and structural barriers, such as health practitioners lacking time, energy, or the ability to be champions; and pervasive discrimination and stereotyping within the health system (Foster, 2017). In other words, there is potential for champions of cultural safety to be change makers, but health organisations and systems must step up too.

To be successful, models of culturally safe maternal–infant care for Māori whānau will require organisational and structural commitment, and the full engagement of the hearts and minds of staff (Balik et al., 2011). These will be hard to achieve for Māori without structural, institutional, and interpersonal racism being addressed and culturally safe care being the norm. Champions are key players in successful implementation science and organisational change due to their ability to facilitate change through commitment, diligence, enthusiasm, and conviction (Bonawitz et al., 2020; Miech et al., 2018). We also see Indigenous patients and whānau as the ultimate experts of what is or is not culturally safe health care. Whānau are, therefore, key to identifying champions, so that the exploration of the neonatal care champions provide can then promote a whānau-centric spread of culturally safe neonatal care and institutional change.

## Methods

### Aim

The aim of this study was to explore the experiences, views, and attributions of health practitioners identified as champions by whānau of preterm Māori infants.

### Methodology

Kaupapa Māori (by Māori, for Māori) research is a response to the deficits-based research that has often been conducted on Māori by non-Māori researchers using Eurocentric inquiry paradigms. Rather than othering Māori, it sees being Māori as normal and promotes structural analyses of health inequities (Cram, 2017; Smith, 2021). The research is led by Māori, for the betterment of Māori – using Māori methods and/or adapting complementary Western methods, for example, the use of photovoice in research with Māori whānau (Jones et al., 2013). In doing so, Kaupapa Māori research supports Māori autonomy over research decision-making and culturally safe research processes that are grounded in Māori ways of being, knowing, doing, and relating (Cram, 2017; Ormond et al., 2006; Smith, 2021). Hence, for this study, the authors/researchers are all Māori and, to ensure the inquiry paradigm was appropriate, were guided by a Kāhui Kaumātua (council of subject expert elders) and a Rōpū Māmā (advisory group of Māori mothers of children born preterm).

As part of the prospective Kaupapa Māori (by Māori, for Māori) qualitative longitudinal research study He Tamariki Kokoti Tau: Babies born prematurely (Adcock et al., 2021, 2022), which explored Māori whānau experiences of preterm care pathways from birth to first birthday, we interviewed 10 health practitioners who were identified by the whānau as champions. In this article, we explore how these health practitioner champions talk about their roles, captured in in-depth semi-structured interviews where they were encouraged to speak freely and to reflect upon their practices. Although this cross-sectional study describes interviews with health practitioners who were not necessarily Māori, their views were sought precisely because they were identified as champions of care by Māori whānau, that is, Indigenous families. This approach, seeking the views of Indigenous family–identified health practitioner champions, is missing from the extant cultural safety literature.

The interviews were analysed with interpretative phenomenological analysis (IPA), which was chosen for its concern with how participants experience and interpret phenomena – in this case, their experiences of supporting whānau of preterm Māori infants (Smith et al., 2009). The researcher’s interpretative activity is also involved in IPA, in what is known as a ‘double hermeneutic’ (Smith et al., 2009). The lead researcher (first author) is a sociologist, experienced in eliciting people’s health-related narratives, and was supported by the co-authors (all senior Māori health researchers, including two clinicians). This exploration of health practitioner champion views can add to the evidence base about culturally safe neonatal care for preterm Māori infants and their whānau. It may also be relevant for advocating for culturally safe championship in other settler-colonial nation-states.

### Ethics

The study received institutional ethical approval from the Northern B Health and Disability Ethics Committee (17/NTB/7, 27/04/2017) and locality approval from the participating District Health Boards. Written informed consent was obtained from all participants, including for the publication of study results.

### Participants

Eleven health practitioner champions identified by seven whānau of preterm Māori infants were approached for an interview (by the first author). ‘Health practitioner’ here denotes any workers involved in the care of the preterm infants and their whānau. Ten health practitioner champions from three hospitals consented to a single interview, either in person at their workplace (*n* = 5) or by telephone (*n* = 5) (1 declined). Two of the health practitioners identified as Māori (eight non-Māori) (other demographic information was not collected). The health practitioners had all supported the whānau in the previous 12 months. Table 1 provides the participants’ unique identifiers, their roles, and if they identified as Māori. To protect their confidentiality, no other information is provided.

**Table 1.** Participant Information.

Identifier	Role	Māori^a^
HP01	Senior medical officer	N
HP02	Neonatal nurse	N
HP03	Neonatal nurse	Y
HP04	Neonatal nurse	N
HP05	Neonatal nurse	N
HP06	Kaiāwhina (non-clinical Māori health worker)	Y
HP07	Neonatal nurse	N
HP08	Counsellor	N
HP09	Neonatal nurse	N
HP10	Neonatal nurse	N

^a^*Note*. Self-identified ethnicity.

### Data Collection

Interviews were conducted between 2017 and 2018. They were in-depth and semi-structured around three topics: involvement, explanations, and whānau coping; with follow-up questions asked to seek clarification or more detail. These are provided in Table 2. This style of interviewing, where the interviewees are able to guide the discussion, is compatible with the chosen method of data analysis – IPA (Smith et al., 2009).

**Table 2.** Interview Questions.

Interview questions	Example follow-up questions
1. What has been your involvement with the infant/s and whānau so far?	• How has that gone?
2. How have you been involved in explaining what has been happening with the infant/s to the whānau?	• Do you feel you were able to communicate what is going on in a way they understood?
3. Do you have any comment about how the whānau have coped with their infant/s’ admission to neonatal care?	• Is there anything that you think could better support whānau coping?

Following time taken for greetings, establishing rapport, and informed consent, the interviews were audio-recorded. The interviews lasted between 17 and 54 minutes. They were transcribed verbatim and participants were given the option of reviewing their transcript.

### Data Analysis

Interpretative phenomenological analysis is concerned with how people make sense of their lives, particularly when experiencing an important event/phenomenon (Smith et al., 2009). It has been used in previous Kaupapa Māori health research studies for its compatibility with Māori understandings of the complexities of experience and relatedness (Jones et al., 2013; Stevenson, Cram, et al., 2020).

In this study, the phenomenon of concern was health practitioner champion experiences of supporting whānau following preterm birth. Each participant (case) transcript was initially analysed separately. The transcripts were annotated (by the first author) with notes about content, linguistic features, and preliminary interpretation (Smith et al., 2009). This initial descriptive coding of the transcripts was then reviewed across transcripts and related themes gathered together to create superordinate and subordinate themes (Bond et al., 2015). The interpretation of the corpus of champion interviews provided a comprehensive picture of their experiences and the meanings they attributed to them (Smith et al., 2009). All stages of the analysis were discussed at regular peer support meetings to provide peer validation of the analysis (Campbell-Jackson et al., 2014).

## Results

**Figure 1.**
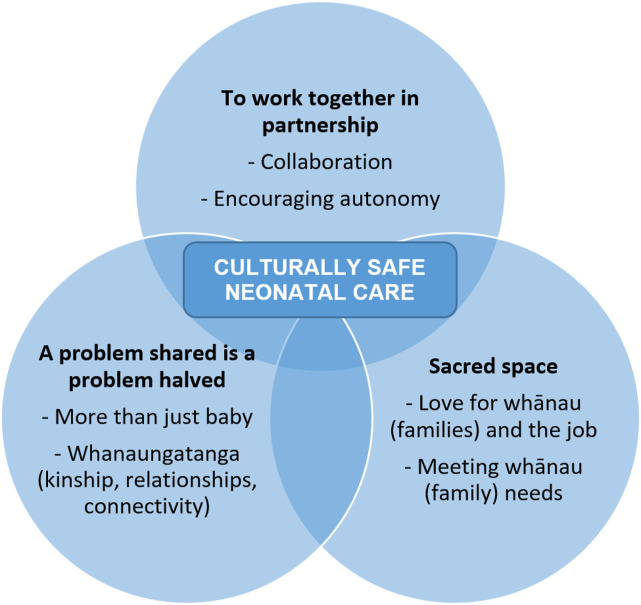
Superordinate and subordinate themes.

Three superordinate themes were created and given their names from participant quotes: *To work together in partnership*, *A problem shared is a problem halved*, and *Sacred space*. Each contains two subordinate themes (see Figure 1), which were present in at least eight interviews (i.e. 80%–100% of interviews). The superordinate and subordinate themes are described below, illustrated with selected participant quotes.

### To Work Together in Partnership

The health practitioner champions all spoke of the importance of collaboration – working together in partnership with their colleagues as well as with whānau. Their roles, whether based within the NICU or supporting health services, necessitated flexibility and collegiality to help whānau navigate the often-unfamiliar phenomenon of preterm birth (and the health services involved). These collaborative relationships were almost always posed as positive and normal practice, although sometimes tensions (between whānau and health practitioners or between health practitioners) were raised. Such tensions were overcome by the health practitioners by privileging whānau voice and actively seeking to problem solve.

#### Collaboration

Health practitioners emphasised the importance of acknowledging if they were not the right person to answer a question or tackle an issue. They would then link whānau to appropriate support, such as a nurse specialist, mentor, doctor, or anyone that might have the right knowledge or resources. Such collaboration was seen as important for providing care to whānau. For example, a neonatal nurse talked about collegiality as a normal part of the job:

*If I felt like I couldn’t explain it, I either went to a colleague or the nurse specialists, or the medical team for them to explain it, so lots of collaboration.* (HP03)

When faced with an issue that could not be easily solved within their team, the health practitioners were creative and flexible, drawing on their wider health service connections to help problem solve. Another neonatal nurse described helping a young couple access a breast pump for expressing milk very late one night, reflecting on the benefits of collaborative relationships:

*‘You need a pump!’ Oh yes, it was 2 o’clock at night, and so I rung up the [maternity unit] and there was a lovely midwife, my God, she was really nice! It's nice when you have colleagues in the other unit and you work together like this.* (HP07)

While collegial relationships were mostly described positively, a tension was revealed for health practitioners who disagree with their peers. For example, a neonatal nurse talked about frictions that can happen when trying to help clarify inconsistencies in messaging for parents:

*At times I do offend and upset other nurses, because I will, from their point of view, I’ll put parents first and not my colleagues first. As in, I don’t have their backs when it comes to their decision.* (HP10)

They emphasised the importance of letting parents and whānau know that there is no one approach to taking care of preterm infants, and that health practitioners will have their own ways of doing things. While they had come up against disappointment from colleagues for this, they said that it was easy for them because they had confidence in themselves and empathy for parents. As such, collaboration with colleagues was presented as important for supporting infants and their whānau, but less so if it was seen to hinder that support.

#### Encouraging Autonomy

The health practitioners consistently advocated for the inclusion of parents in the day-to-day cares of their infants, noting that all whānau are capable. They talked about the importance of keeping parents informed of progress and changes and teaching them how to care for their preterm infant. Strategies, such as asking open questions or using the teach-back method to check understanding, were described. It was important for the health practitioners to pitch information at the right level, which meant getting to know each whānau. For example, a neonatal nurse talked about gauging health literacy. This was conveyed in a way that put the responsibility for explanations on the health practitioner rather than perpetuating deficit views about health illiteracy:

*I just think it’s important that you gauge their health literacy level and make sure all your explanations are communicated in a way that they'll understand.* (HP04)

The health practitioners reflected on learning from parents and whānau, acknowledging that listening is crucial. They noted that parents will know their infant well from spending dedicated time with them and will often be the first to notice if something is wrong. Listening was posed as engendering relationship building and showing parents respect. A neonatal nurse articulated the need to encourage this:

*I identify with them and if I can see there’s an unwillingness, perhaps because they're shy or they think that they’re not allowed to, I can explain the situation to them and encourage them in their autonomy and in their independence. And say, you know, ‘It's good for you to ask these questions, it’s good for you to know and have the answers, and have input and say.’ Because the medical team is one side, but the parents know the baby in a way that we don’t.* (HP10)

This was a recurring sentiment – that parents and whānau are integral to infant care and they should be informed and involved in decision-making and cares. Mutual trust was positioned as crucial for working in partnership with whānau. The health practitioners suggested that when whānau are not intimidated by them, there is a better chance of reaching an understanding after any disagreements or tensions. A counsellor spoke of the negotiated partnership between mothers and nurses in the NICU, describing it as like a dance in which roles are established – where mothers are strengthened in their roles as mothers and the main care providers, and health practitioners in their roles as health care providers:

*A lot of the mums really struggle with feeling like this is not their baby, and that's a huge thing that comes up, that this doesn't feel like my baby, that I have to ask permission. And so, it’s that whole dance that nurses do with mums **to work together in partnership**.* (HP08)

The health practitioners frequently lamented the struggles they saw in parents who felt disconnected from their infants due to the NICU routines and environment. As holistic approaches to neonatal care, FCC, FiCare, and patient-centred care were presented as good opportunities to encourage whānau autonomy.

### A Problem Shared Is a Problem Halved

The health practitioners all identified the role they played in establishing relationships between themselves and whānau. They described this connectivity as a form of critical social support for whānau of preterm Māori infants while they were in the NICU. Not only was the strengthening of these relationships a key way of supporting the emotional wellbeing of whānau, the health practitioners described experiences of it positively impacting medical outcomes. Caring for parents and creating space for them to connect with whānau and with peers was presented by the health practitioners as relieving some of the burden of infant hospitalisation.

#### More Than Just Baby

Developing rapport through reassuring, comforting, and empowering parents was emphasised by the health practitioners as crucial for the health and wellbeing of the whole whānau. The health practitioners’ care roles extended to spending time with whānau, especially parents, to engage with them personally and ensure that they felt that they were appreciated and cared for. A neonatal nurse described their role as being more than just infant care; they provided this care within a conversational, friendly context:

*I know we’re there to look after baby, but it’s having conversations that aren't even related to baby and baby’s care and things like that, talking about how their weekend is going and what they've been up to.* (HP04)

Such caring was noted as being particularly significant for fathers, who would often feel more alienated due to spending less time in the NICU because of other responsibilities (e.g. employment and other children). Everyday conversations were seen as promoting a sense of normalcy for parents in the abnormal environment of the NICU, with familiarity a comfort:

*I become really involved in teaching the family because I’m quite a consistent person... So, four days out of every seven the people see me every day, and so I become, I guess I become like this familiar figure.* (HP2)

This was also talked about as continuity of care:

*I think that's really important — that continuity of care, not only for baby but for mums as well, or for the whole family really.* (HP09)

Such care was positioned as requiring staff to be attentive and proactive, taking note of the individual context of each whānau, and following up to ensure issues are resolved. Ensuring that support is offered more than once – when whānau might feel uncomfortable accepting it at first or if situations change – was also noted as important. For these champions, there was consistent recognition that their role was about more than the preterm infant patient; it was about caring for a whole whānau.

#### Whanaungatanga (Kinship, Relationships, and Connectivity)

Derived from the word ‘whānau’, whanaungatanga can mean a sense of kinship, relationships, and connectivity. Understanding the importance of whānau and whanaungatanga for parents of preterm Māori infants was advocated for by the champions. Involving whānau, as in extended family collectives, in caring for parents and their preterm infants was thought to benefit the establishment of loving and supportive routines that may then continue when infants are discharged home. For example, a senior medical officer said that their personal policy was to include whānau in discussions and decision-making:

*I understand the importance of whānau and how we need to involve them, and so I ask people, ‘Do you need anyone here, your extended whānau?’ If they do, and if they want me to meet with them, then I’ll meet with them. If they don’t, I won’t. So, I’ll give the choice to them.* (HP01)

For those mothers/parents who experience a transfer from their home region, away from whānau, the health practitioners acknowledged the unique challenges faced:

*The hardest is when the family is, or the baby gets born and planed here... When all the family is in the [other island] it's quite hard on families, because then they can't come down all the time, and things have to be communicated over the telephone. That's not the same, it's not the same emotional support when somebody needs a hug, when somebody needs just to hold somebody.* (HP07)

In these situations, a sense of connection for parents was stressed as particularly important. Peer support, by other parents in the NICU, was proffered as a way of developing such connections (whether parents are transferred or not). A counsellor used a common idiom to describe the benefits of this:

*Talking with other parents, they saw that there were other people going through similar things as them. What’s that saying? **A problem shared is a problem halved** kind of thing, and I think that probably helped them.* (HP02)

The whānau they had been supporting had been transferred to a NICU in a different region, and the counsellor thought that meeting and sharing with other whānau in the same situation had helped them to cope. In the absence of kin, peers and other supporters can provide much needed care.

### Sacred Space

The health practitioners acknowledged the special – and sacred – time that childbirth is and the trauma that preterm birth causes for whānau. They appreciated their important role in supporting whānau to overcome challenges and spoke of love and feeling privileged. Aspirations for a future where the health services that they work in better accommodate whānau (in all senses of the term accommodate) were shared, as were actions that these health practitioners take in their day-to-day work to move towards these goals.

#### Love for Whānau (Families) and the Job

The health practitioners spoke of love and passion for their job and the whānau they support. They described their work as a privilege. Framing their work as joyful and special rather than frustrating or mundane, even though some health practitioners spoke of having to deal with understaffing or other pressures, gave the impression that love – genuine compassion, empathy, and care – is a vital component for delivering good care. For example, a senior medical officer thought that they had the best job in the world:

*And the joy we get out of making them better, or helping them get better, you can't have anywhere else.* (HP01)

They said they thrived off the faith that parents have in them and the reciprocal trust they build, not wanting to imagine working in any other field. Similarly, a neonatal nurse talked about passion and love for their job and spending time with whānau:

*I’m pretty passionate about it to be honest, and I, like, love it! Yeah, I think it would be quite horrible for them to sit there and listen to me, ‘Oh I wanna go home.’ Like, I love being here.* (HP05)

Their recognition that seeming disinterested in their work would impact negatively on parents highlights the importance of genuine compassion and care. Care and understanding of the significance of childbirth was also spoken of in spiritual terms. A kaiāwhina expressed a deep appreciation for being welcomed into whānau spaces, especially the sacred space of birth:

*It is a lovely role. It's quite a special time because birth and death is such a **sacred space**, and so to be a part of that space is very very sacred and special, and it is a taonga [precious thing]. And each time I’m allowed in that space of that whānau and the neonatal [unit] prior to birth and after, is such a privilege, it is such a privilege… You can encourage, have some input in that baby’s wellbeing for their future.* (HP06)

This kaiāwhina worked to ensure that other non-Māori health practitioners caring for whānau in the hospital were aware of this sacred space and the importance of being responsive, by mediating issues and assisting with te reo Māori (Māori language) name pronunciation and basic understanding. They supported whānau by helping them to develop holistic health plans that followed Māori health models. They connected whānau with Māori specific support services and assisted whānau with Māori cultural protocols, such as karakia (prayer/incantations) and waiata (songs/singing).

#### Meeting Whānau (Family) Needs

The health practitioners reflected on NICU realities for whānau and ventured aspirations for improvements to neonatal health services. As already discussed, the importance of whānau was frequently emphasised by the health practitioners, but in trying to meet the needs of whānau, they would come up against unit policies and resourcing issues. Neonatal intensive care unit visiting hours and rules, such as who can visit and how many at a time, were presented as challenging for whānau, especially for those who have other young children, and even more so if they have been transferred from a rural or different region. A lack of in-hospital or motel accommodation options for larger groups, and sometimes fathers as well, was noted.

Neonatal intensive care unit restrictions were rationalised as protecting the infant from too much stimulation because of limited physical space. However, it was suggested that more private spaces for whānau, and better communication to whānau about restrictions, would be beneficial. Sometimes, the health practitioners would be creative with how they worked around these restrictions or accommodated whānau, for example, relaxing the rules when possible, to allow important protocols like karakia to take place:

*We don’t have the facilities to accommodate large family groups either, so it's really difficult. But I always try, you know, if families want to come in and have karakia or anything like that, then you know what, that's really important, like sometimes you can relax the rules a little bit.* (HP09)

Another way of accommodating whānau was encouraging the correct use of te reo Māori. The development of more te reo Māori NICU resources and the normalisation of te reo Māori in the hospital were suggested. A counsellor talked about their personal mission to learn te reo Māori:

*But I guess it's just like little things, isn't it? And like for myself, this year I’ve just started to learn te reo Māori, because for me I just realised, you know, language is one of the most important things, isn't it? And if I can, I’ll make an effort and try and make somebody feel more comfortable.* (HP08)

They recognised the importance of language for connecting with people and creating a comfortable environment. Specific Māori support services were also deemed a critical part of ensuring whānau receive appropriate care. These roles would usually be undertaken by kaiāwhina, Kaumātua (Elders), Māori social workers, or Māori nurses. However, the availability of such personnel would be dependent on (often limited) resourcing:

*The Māori health nurses do try and come and see all the patients, but sometimes they can’t get round to it, ‘cause there’s like two of them for the whole hospital.* (HP04)

The health practitioners acknowledged how strained Māori support services were in the mainstream hospital settings they worked in, and the subsequent burden this placed on whānau. They agreed that the responsibility for ensuring culturally safe care for whānau is on everyone.

## Discussion

Our study has highlighted the role of predominately non-Māori health practitioner champions and their ability to advocate for and support whānau during their experience of NICU. When the anticipated birth journeys of the whānau went off-course, because their infants were born prematurely and needed to be admitted to NICUs, whānau were clearly able to identify the health practitioners who were their key supports, their advocates, and their champions. This is a key element of cultural safety, namely, that patients define whether or not their care has been culturally safe (Curtis et al., 2019; Ramsden, 2015).

With the exception of the kaiāwhina, whose role was to help whānau navigate their hospital journeys and support culturally safe care, these health practitioners were not champions of cultural safety in any official capacity. They came from different backgrounds, professions, and locations, yet articulated shared understandings of what is important in terms of caring for whānau of preterm Māori infants. The values and practices the health practitioner champions all brought to the provision of this care can inform the cultural safety training for health practitioners that has been identified as urgently needed if current inequities in adverse perinatal outcomes for Māori are to be eliminated (PMMRC, 2021, 2022). Health services can learn from these champions about values and practices that build and strengthen relationships between health practitioners and whānau.

The health practitioner champions talked about encouraging whānau autonomy and engagement and checking understanding using effective communication strategies. In the provision of care, they exemplified the attributes of champions detailed in wider implementation literature (Bonawitz et al., 2020). They also described, demonstrated, and highlighted aroha (love) – characterising their work as a privilege, joyful, and something they feel love for – appreciating the sacred space that childbirth is, and valuing Māori cultural values and protocols. This affection had been felt by the whānau who identified them as champions. And these findings echo what the whānau said themselves – that culturally safe care is affirming, inclusive, and loving, with health practitioner champions becoming ‘like whānau’ (Adcock et.al., 2021).

From a Māori worldview, in which the world is relationships, aroha is essential to survival and wellbeing (Pere, 1997). ‘Aroha ki te tāngata’ (love for the people) is a key value and practice (Smith, 2021). We postulate that aroha is, therefore, a necessary component of culturally safe care for Māori whānau. Neonatal intensive care is intense, stressful, and abnormal, exposing people to adverse situations. Despite this, the health practitioner champions drew on consistent themes of practice. They did not need costly intensive care ‘gear’ to practise cultural safety, showing that relationships are the safe care foundations, which in ever-stretched health service settings are free. Good communication, sharing, showing love, and holding space in the intensive care environment provide the foundation for systemic change and improved outcomes for Māori (and then all). This is what Māori interviewed in a range of health consumer research projects, over a number of years, have said they want from health practitioners (Palmer et al., 2019).

The health practitioner champions expressed concerns about the isolation and feelings of powerlessness that parents can feel in the NICU and the marginalisation caused when health practitioners take over the care of preterm Māori infants. These concerns were a motivator for the actions taken by the health practitioners to support and alleviate the stress whānau felt, while at the same time, they carefully negotiated with the whānau to establish their roles in a partnership way – so as not to work based on their assumptions about what whānau might need. Their analysis of the ‘problems’ faced by whānau in NICUs is supported by research undertaken with whānau themselves (Adcock et al., 2021, 2022; Māori and Indigenous Analysis Ltd. & Rautaki Ltd, 2010; Stevenson, Cram, et al., 2020; Thompson, 2009), demonstrating how they are in touch with the lived realities of whānau.

The recognition of the stress and disempowerment of NICU admissions by the health practitioner champions has an interface with structural, institutional, and interpersonal racism. These all occur in the current health system and perpetuate the adverse outcomes experienced by Māori (Cormack et al., 2018; Graham & Masters-Awatere, 2020; Thayer et al., 2019). Recognition and action to address racism in all its forms within perinatal health care settings are needed (Dawson et al., 2022). While this study found that culturally safe care is being practiced, the small number of identified champions demonstrates that there is a need for the wider perinatal workforce to work in a culturally safe manner with whānau. Given the limited number of Māori in the health workforce currently, non-Māori health practitioner champions are well placed to support their colleagues and the whānau they care for.

Holistic culturally safe models of care provide the opportunity to transform the current system. Family-centred care and FiCare are considered important in the neonatal context (Gooding et al., 2011; Patel et al., 2018), but not including Indigenous input is a missed opportunity. These approaches need to be culturally safe, taking into account the needs and aspirations of Indigenous peoples – who may have different ideas about what whānau is, including the recognition of more-than-human relations (Ormond et al., 2006). This requires tackling epistemic injustices that privilege European worldviews and knowledge systems (Stevenson, Filoche, et al., 2020). We agree with Foster (2017) that the foundation of cultural safety is good relationships between health practitioners and patients/families, and that this is heart and mind work (Balik et al., 2011).

The dedicated collaborative practices with colleagues and whānau; genuine love and care; and recognition and respect of culture, described by the participants in this study, indicate that Stevenson and colleagues’ (2020) maternal–infant care framework Te Hā o Whānau is not far-fetched and is in keeping with other calls to action (Foster, 2017; Hickey et al., 2021). Whānau – involving family collectives that extend beyond the household – and whanaungatanga – finding a sense of kinship or connectedness – were highlighted in our study as alleviating the burden and isolation of parenting in neonatal intensive care. These need to be viewed as an intrinsic part of evidence-based medical care.

### Limitations and Future Research

This qualitative research study sought the perspectives of Indigenous family–identified champions of culturally safe neonatal care. A small number of health practitioners were interviewed and the findings may not be generalisable, but the depth of their consideration for whānau and the agreement (80%–100%) across the champions strongly suggests that this study has important implications for the cultural safety training called for by the PMMRC (2021, 2022) so that championship can be scaled up as a change agent for improving whānau experiences.

Further research could explore in more depth the systemic barriers experienced by cultural safety champions, along with their own stories of becoming champions and their understanding of the historical, colonial roots of current Māori health inequities.

## Conclusion

This is the first study to explore the perspectives of health practitioners who have been identified as champions of care by Indigenous families in the neonatal context. Who better to say that a health practitioner is a champion for whānau than whānau themselves? Ten such champions spoke in heartfelt ways of their practice models of caring for and supporting whānau whose infants were admitted to a NICU. Champions were key connectors between health care teams and whānau, striving to work in partnership within a sacred space of birth and new life. They navigated, advocated, shared knowledge, and wrapped whānau with support and care. They undertook ‘mahi aroha’ – work done out of a love for the people.

Implications of these findings for health service delivery are both values-based and practice-based, where mahi aroha is an essential component. Indigenous peoples are not homogenous, comprising of distinct nations with unique cultures. However, key messages about cultural safety in neonatal care from this research may be relevant to other high-resource settler-colonial nation-states. They are also applicable to other areas of health care, such as paediatric intensive care and maternity care, where there are many calls for culturally safe health services for Indigenous families.
